# Functional profiles of Müllerian inhibiting substance/anti-Müllerian hormone (MIS/AMH) in primarily cultured endometrial cancer cells

**DOI:** 10.7150/jca.60700

**Published:** 2021-08-28

**Authors:** Sang Il Kim, Joo Hee Yoon, Soo Young Hur

**Affiliations:** 1 Department of Obstetrics and Gynecology, St. Vincent's hospital, College of Medicine, The Catholic University of Korea, Seoul, Republic of Korea; 2 Department of Obstetrics and Gynecology, Seoul St. Mary's hospital, College of Medicine, The Catholic University of Korea, Seoul, Republic of Korea

**Keywords:** anti-Müllerian hormone, Müllerian inhibiting substance, endometrial cancer, Wnt signaling pathway

## Abstract

**Background:** Müllerian inhibiting substance/anti-Müllerian hormone (MIS/AMH) inhibits proliferation of MIS/AMH receptor-expressing gynecologic tumors* in vivo* and *in vitro,* but the underlying mechanisms have not been fully defined. This study aimed to investigate the expression of MIS/AMH type II receptor (MIS/AMHRII) in endometrial cancer, to identify the mechanism of growth inhibition in MIS/AMH-treated endometrial cancer cells, and to evaluate the clinical significance of MIS/AMH as an effective targeted therapy for MIS/AMH receptor-expressing tumors.

**Methods:** We used tissue samples from 10 patients with total hysterectomy for endometrial cancer. To identify involved signaling pathways, we performed western blotting on apoptosis-, cell cycle-, Wnt signaling-, and autophagy-related proteins.

**Results:** MIS/AMHRII was highly expressed on the cell membrane of endometrial cancer tissues and primarily cultured endometrial cancer cells. We also found that MIS/AMH treatment reduced cell viability, induced cell cycle arrest, and increased apoptosis. MIS/AMH treatment induced upregulation of β-catenin-interacting protein (ICAT) and inhibition of the Dvl and Axin complex (IDAX) but downregulation of phospho-c-Jun in the Wnt signaling pathway.

**Conclusions:** MIS/AMH inhibits the growth of MIS/AMH receptor-expressing endometrial cancer cells through regulation of autophagy, apoptosis, and cell cycle pathways, as well as inhibition of Wnt signaling pathways. These data suggest that MIS/AMH functions as a tumor suppressor and may be an effective therapeutic agent in endometrial cancer.

## Introduction

The incidence of endometrial cancer (EC) has been increasing, and it is now the most common gynecologic cancer in Korea. In 2020, approximately 3,261 new cases and 383 deaths were expected [1.2]. Treatment options for EC are generally limited to surgery, chemotherapy, and radiation therapy. Although early-stage EC is associated with favorable survival outcomes, no cure exists for advanced, recurrent, or metastatic EC, which has only limited treatment options. In the United States, it is one of the cancers with an increasing incidence and mortality 3. Thus, novel agents that improve oncologic outcomes are needed.

Müllerian inhibiting substance (MIS), known as anti-Müllerian hormone (AMH), was first discovered and reported by Alfred Jost in 1947 4. MIS/AMH is produced in Sertoli cells of the fetal testis and binds to MIS/AMH receptors to cause in male embryos the regression of Müllerian ducts, which are precursors of the fallopian tubes, surface epithelium of the ovaries, uterus, cervix, and upper third of the vagina. MIS/AMH is a member of the TGF-β superfamily of growth factors and a 140-kDa dimeric glycoprotein composed of two identical disulfide-linked subunits of 535 amino acids. The receptor is a heteromeric complex consisting of type I (MIS/AMHRI) and type II (MIS/AMHRII) transmembrane serine-threonine kinase receptors. The inhibitory function of MIS/AMH begins with ligand binding to the primary receptor, MIS/AMHRII. Then, MIS/AMHRI is recruited into the receptor complex, resulting in the activation of MIS/AMHRI via cross-phosphorylation by MIS/AMHRII, activating intracellular signaling cascades 5.

In addition to its role in fetal sexual development, MIS/AMH has been extensively studied as a biological marker of ovarian function and reserve. Several studies have shown that MIS/AMH is significantly associated with cycle irregularity and the onset of menopausal transition. Since MIS/AMH is the earliest marker to decrease with age and has the least intercycle and intracycle variability, it has strong potential to be used as a conventional marker for ovarian reserve. In addition, higher MIS/AMH serum levels are associated with *in vitro* fertilization outcomes, such as the number of retrieved oocytes, number of fertilized oocytes, and clinical pregnancy rate 6, 7.

Furthermore, MIS/AMH has been extensively studied as a diagnostic marker and therapeutic agent for gynecologic cancer. MIS/AMH has only been shown to be a marker for granulosa cell tumors. Serum MIS/AMH normalizes with successful treatment and increases early with disease recurrence, indicating that MIS/AMH could be used to assess treatment efficacy 8. Since most gynecologic tumors originate from the Müllerian duct and MIS/AMH causes regression of the Müllerian duct, MIS/AMH should inhibit the growth of gynecologic tumors. Purified recombinant human MIS/AMH causes growth inhibition of epithelial ovarian cancer cells and cell lines *in vivo* and *in vitro* via MIS/AMH receptor-mediated mechanisms 9-13. Several studies have suggested that MIS/AMH inhibits growth in tissues and cell lines of other MIS/AMH receptor-expressing gynecologic tumors, such as cervical, endometrial, and breast cancers, as well as endometriosis, but its downstream regulatory signaling pathways have not been fully elucidated 14-19. In previous studies, cell cycle arrest has been regarded as a major factor in MIS/AMH-mediated signal transduction cascades in gynecologic cancer. MIS/AMH upregulates the expression of p16, pRB-related proteins, and some E2F family members, and induces G_1_ arrest and subsequent apoptosis, but gene regulation by MIS/AMH appears to be specific to the tumor type 9, 14-16, 20. The Wnt signaling pathway also plays an important role in both embryonic development and tumorigenesis. β-catenin, a key component of the Wnt signaling pathway, interacts with the T-cell factor (TCF)/lymphoid enhancer factor (LEF) family of transcription factors and activates the transcription of Wnt target genes, which in turn regulate proliferation, polarity, adhesion, and motility 21, 22. This was also demonstrated in our previous study. The endometrial cancer cell line AN3CA, which strongly expresses MIS/AMHRII when treated with MIS/AMH, upregulates the β-catenin-interacting protein (ICAT), which negatively regulates the Wnt signaling pathway by inhibiting the interaction between β-catenin and TCF family members 17. MIS/AMH upregulates ICAT expression, which inhibits the growth of the human epithelial ovarian cancer cell line OVCAR-8 by disrupting the β-catenin-dependent Wnt signaling pathway. ICAT downregulation by small interfering RNAs (siRNA) reverses the effects of MIS/AMH, which decreases cell viability and migration while promoting apoptosis 13.

Thus, this study aimed to investigate the expression of MIS/AMH type II receptors in patient-derived endometrial cancer cells to identify the mechanism of growth inhibition in MIS/AMH-treated endometrial cancer cells and to determine whether MIS/AMH administration could be an effective targeted therapy for MIS/AMH receptor-expressing tumors.

## Materials and Methods

### Study Objectives

We included three premenopausal and seven postmenopausal women (average age, 55.7 years) who underwent total hysterectomy for endometrial cancer from July 2012 to April 2015 at Seoul St. Mary's Hospital. The endometrial cancer was categorized by two experienced pathologists as 4 well-differentiated endometrioid adenocarcinomas, 4 moderately differentiated endometrioid adenocarcinomas, and 2 poorly differentiated endometrioid adenocarcinomas. Dissected tissue samples were fixed in 4% paraformaldehyde solution and embedded in paraffin.

This study was approved by the Institutional Review Board/Human Research Committee of the Seoul St. Mary's Hospital (# KC11TIS0576). The study was conducted in accordance with the Declaration of Helsinki.

### Immunohistochemistry

Paraffin-embedded tissue was sliced to a thickness of 5 µm and attached to ProbeOn Plus slides (Fisher Scientific, Pittsburg, PA, USA). Tissues for immunohistochemical detection of MIS/AMHRII were processed using the capillary tube method with a fast temperature-controlled machine and a microprobe immunostaining station (Biomeda Co., Foster City, CA, USA). The immunostaining procedure was as follows: the slides were deparaffinized with xylene (5 min, 3 times) at room temperature, washed with 100% alcohol (5 min, 3 times) to remove xylene, then rehydrated with water. To retrieve antigenic sites, the slides were incubated at 121°C for 10 min in citrate buffer (Zymed Laboratories Inc., South San Francisco, CA, USA) and cooled for 20 min at room temperature. The slides were then treated with 3% hydrogen peroxide for 10 min to eliminate endogenous peroxidase activity followed by washing with phosphate-buffered saline (PBS) 4 times. After washing with PBS, slides were immunostained using a CAP-PLUS detection kit (Zymed 87-8143; Zymed Laboratories Inc.). After treatment with 10% normal goat serum for 1 h to block non-specific protein binding followed by washing with PBS, the slides were incubated with rabbit polyclonal anti-human MIS/AMHRII antiserum (153P; provided by Professor Patricia K. Donahoe, Massachusetts General Hospital, Boston, MA, USA) as the primary antiserum at 4°C overnight. The slides were rinsed in PBS (5 min, 3 times) and incubated with biotinylated anti-rabbit IgG (Zymed Laboratories Inc.) as the secondary antibody at room temperature for 1 h. After rinsing with PBS, a streptavidin HRP detection system (Zymed Laboratories Inc.) was applied to the slides for 30 min to induce the biotin-avidin binding reaction. After being washed with PBS for 5 min, the slides were treated with 3-amino-9-ethylcarbazole (AEC) for 10 min at room temperature, counterstained with hematoxylin, and then mounted with glycerol gel.

### *In situ* Hybridization

#### Generation of the RNA Probes for the MIS type II Receptor

To obtain the *in situ* RNA probe, a 361-bp PCR product was prepared from the full-length human MIS/AMHRII cDNA (provided by Professor Patricia K. Donahoe) using the primers (Gene Bank, Accession No. AF172932; upstream primer, sequence 581-600 and downstream primer, sequence 921-941) and cloned into the T-Easy vector (Promega Corp., Madison, WI, USA). The digoxigenin (DIG)-labeled sense and antisense human MIS/AMHRII RNA probes were prepared by *in vitro* transcription using a DIG RNA Labeling Kit (Boehringer Ingelheim GmbH, Mannheim, Germany) according to the manufacturer's protocol. The titer of the RNA probe was examined using the dot blot method.

#### *In situ* Hybridization

Tetramethylbenzidine (TMB) sections of 5 µm thickness were preheated at 85°C in an oven for 30 min. After dewaxing with xylene (2 min, 4 times), the slides were rinsed with 100% alcohol and dried at 40°C in an oven for 1 h. The dried sections were treated in 0.2 N HCI for 20 min and incubated in 20 µg/ml pepsin (0.1 N HCI) for 20 min at room temperature, followed by three washes with diethylpyrocarbonate-treated PBS. The sections were dehydrated in ethanol and dried. Prehybridization and hybridization steps were carried out at 53°C for 2 h and 15 h, respectively. The prehybridization buffer was composed of 50% formamide, 4× saline-sodium citrate (SSC), 10% dextran sulfate, 1× Denhardt's solution, and 1 mg/ml salmon sperm DNA. The hybridization buffer was identical to the prehybridization buffer, except that salmon sperm DNA was substituted with 200 ng/ml MIS/AMHRII riboprobe. After post-hybridization washing, sections were incubated with anti-digoxigenin antiserum conjugated with alkaline phosphatase (Boehringer Ingelheim GmbH), and histochemical detection was then performed using 4-nitro blue tetrazolium and 5-bromo-4-chloro-3-indolyl-phosphate (Boehringer Ingelheim GmbH).

### Endometrial Cancer Cell Culture

Fresh human endometrial cancer specimens, collected from five patients who had undergone total hysterectomy for endometrial cancer, were processed as quickly as possible. The tissues were rinsed twice with PBS and cut into 1-3 mm^3^ pieces. Portions of the minced tumor were then placed into 250 ml flasks containing 30 ml of enzyme solution (0.14% collagenase type I and 0.01% DNase [2000 kU/mg]; both Sigma-Aldrich, St. Louis, MO, USA) in DMEM*/*F-12 (Gibco*-*BRL, Grand Island, NY, USA), and incubated on a magnetic stirring apparatus for 2 h at 37°C. The enzymatically dissociated tumor was then filtered through a 100-mm nylon mesh to generate a single-cell suspension. The resultant cell suspension was washed twice and incubated in DMEM*/*F-12 (Gibco-BRL) containing 10% fetal bovine serum, 100 U/ml penicillin, and 100 U/ml streptomycin at 37°C in humid air with 5% CO_2_.

### Immunocytochemistry of Cultured Endometrial Cancer Cells

MIS/AMHRII expression was immunocytochemically detected using the Invitrogen Histostain Plus AEC kit (Invitrogen, Carlsbad, CA, USA) according to the manufacturer's instructions. Cultured human endometrial cancer cells were harvested, 200 µl of a 1×10^5^ cells/ml suspension was centrifuged with a cytospin (Thermo Electron Corp., Cheshire, WA7, UK) at 1,000 rpm for 5 min, and the cell pellet was transferred to ProbeOn Plus slides (Fisher Scientific). These slides were then treated with 3% hydrogen peroxide for 5 min to eliminate endogenous peroxidase activity and washed three times with PBS. After treatment with donkey serum (Invitrogen) for 30 min to block non-specific protein binding, the slides were incubated with rabbit polyclonal anti-human MIS/AMHRII antiserum (153P) at 4°C overnight. The slides were rinsed in PBS three times and incubated with biotinylated anti-rabbit IgG (Invitrogen) for 30 min. After another three PBS rinses, the streptavidin HRP detection system (Invitrogen) was applied to the slides for 30 min to induce the biotin-avidin binding reaction. The slides were treated with AEC for 10 min at room temperature, counterstained with hematoxylin, and mounted with glycerol gel. Three hundred endometrial cancer cells were counted in five different microscopic fields, and the mean percentage of immunopositive cells was calculated.

### MIS/AMH expression and purification

Recombinant human MIS/AMH was expressed in dihydrofolate reductase-deficient Chinese hamster ovary cells and secreted into chemically defined serum-free media. The serum-free media was collected every 48 to 72 hours and purified by antibody affinity chromatography using a monoclonal antibody to MIS/AMH. The mouse monoclonal antibody used for this column is highly specific for human MIS/AMH and does not interact with other members of the TGF-β family of proteins. Cleaved MIS/AMH was generated by digesting full-length MIS/AMH with plasmin at a mass ratio of 25:1 for 90 min at room temperature. In order to purify carboxy-terminal MIS/AMH from cleaved MIS/AMH, the pH of the plasmin-treated sample was reduced to pH 3.5 with acetic acid. The mixture was then loaded onto wheat germ lectin column equilibrated in 25 m*M* glycine 0.5*M* acetic acid, pH 3.5 to bind the carbohydrate containing amino-terminus. Pure MIS/AMH mature fractions were confirmed by SDS-PAGE 23.

### Methylthiazoletetrazolium (MTT) Assay

Three thousand cells per well were seeded into 96-well plates. After 24 h, the cells were exposed to vehicle control or 10 µg/ml of MIS/AMH (provided by Professor Patricia K. Donahoe) for 24, 48, or 72 h. Cells were washed with PBS, and 100 µl of MTT solution (5 mg/ml MTT stock in PBS diluted to 1 mg/ml with 10% DMEM) was added to each well. Cells were incubated for 4 h at 37°C after which 200 µl DMSO (Sigma-Aldrich) was added and incubated further for 30 min at room temperature in the dark. Optical densities at 550 nm were measured using an ELISA plate reader (BIO-TEK Instruments, Winooski, VT, USA).

### Cell Cycle Analysis

Cells were exposed to medium with 10% fetal bovine serum and 10 µg/ml MIS/AMH for 72 h and collected afterward, following trypsinization. The cells were fixed with 100% methanol, stored for 30 min at 20°C, and washed with PBS. Following centrifugation, the cells were resuspended in 1 ml DNA staining solution (20 µg/ml propidium iodide, 200 µg/ml DNase-free RNase) and incubated in the dark at 37°C for 30 min. The cells were analyzed using a FACSVantage SE Flow Cytometer (Becton Dickinson, San Jose, CA, USA). Forward scatter and red fluorescence above 600 nm were measured, and the results were analyzed using CellQuest^TM^ software (Verity Software House, Topsham, ME, USA).

### Annexin V Analysis

MIS/AMH-treated cells were stained with annexin V and 7-amino-actinomycin (7-AAD) using the Annexin V:PE Apoptosis Detection Kit I (BD Biosciences, San Diego, CA, USA) according to the manufacturer's protocol. Briefly, following drug treatment, 1×10^5^ cells were pelleted, washed once with PBS, and resuspended in 100 ml of binding buffer (10 mM HEPES [pH 7.4], 150 mM NaCl, 5 mM KCl, 1 mM MgCl_2_, and 2 mM CaCl_2_). Subsequently, 5 µl of annexin V-PE and 7-AAD was added to the cells, which were then incubated for 15 min at room temperature in the dark. After incubation, 400 µl of binding buffer was added, and cells were analyzed using a FACSVantage SE Flow Cytometer (Becton Dickinson). Data analyses were conducted using CellQuest^TM^ software.

### Western Blot Analysis

Proteins from cells treated with 10 µg/ml MIS/AMH were harvested in RIPA buffer (150 mM NaCl, 1% NP-40, 0.5% sodium deoxycholate, 0.1% SDS, 50 nM Tris-HCl) with 1 μM PMSF, and the protein concentration was determined using BCA protein assay reagent (Thermo Scientific, Waltham, MA, USA). Equal amounts of protein were separated on SDS-polyacrylamide gels (50 µg per lane) and transferred to PVDF membranes. The blots were blocked for 1 h in TBS-T (20 mM Tris-HCl [pH 7.6], 137 mM NaCl, 0.1% Tween-20) containing 5% powdered milk and then incubated in 1% milk TBS-T with the primary antibodies at 4°C overnight. Antibodies against apoptotic prote; Santa Cruz Biotechnology, Santa Cruz, CA, USA), Caspase-3 (9668; Cell Signaling Technology, Inc., Boston, MA, USA), PARP (9542; Cell Signaling Technology, Inc.), cyclin dependent kinase 2 (CDK2; sc-6248*;* Santa Cruz Biotechnology), p130 (sc-317; Santa Cruz Biotechnology), p107 (sc-318; Santa Cruz Biotechnology), ICAT (sc-99240; Santa Cruz Biotechnology), inhibition of the Dvl and Axin complex (IDAX; sc-164631; Santa Cruz Biotechnology), phospho-c-Jun (3270; Cell Signaling Technology, Inc.), beclin-1 (3495; Cell Signaling Technology, Inc.), and LC3A/B (12741; Cell Signaling Technology, Inc.) were used at 1:200 dilution. Blots were then washed three times with 1% milk TBS-T and incubated with the corresponding horseradish peroxidase-conjugated secondary antibody. Blots were detected using Pierce ECL Western Blotting Substrate (Thermo Scientific).

### Expression Scoring System and Statistical Analysis

The intensities of immunohistochemistry and *in situ* hybridization stainings were assessed independently by two pathologists using a scale of increasing intensity: 0 (no staining), 1 (weak), 2 (moderate), and 3 (strong staining). Statistical comparisons between two experimental groups were performed using the paired Student's *t*-test, whereas multiple group comparisons were performed using analysis of variance. Data were considered significant when *P*<0.05.

## Results

### Immunohistochemical Detection of MIS/AMHRII Protein Expression

**Figure 1:**
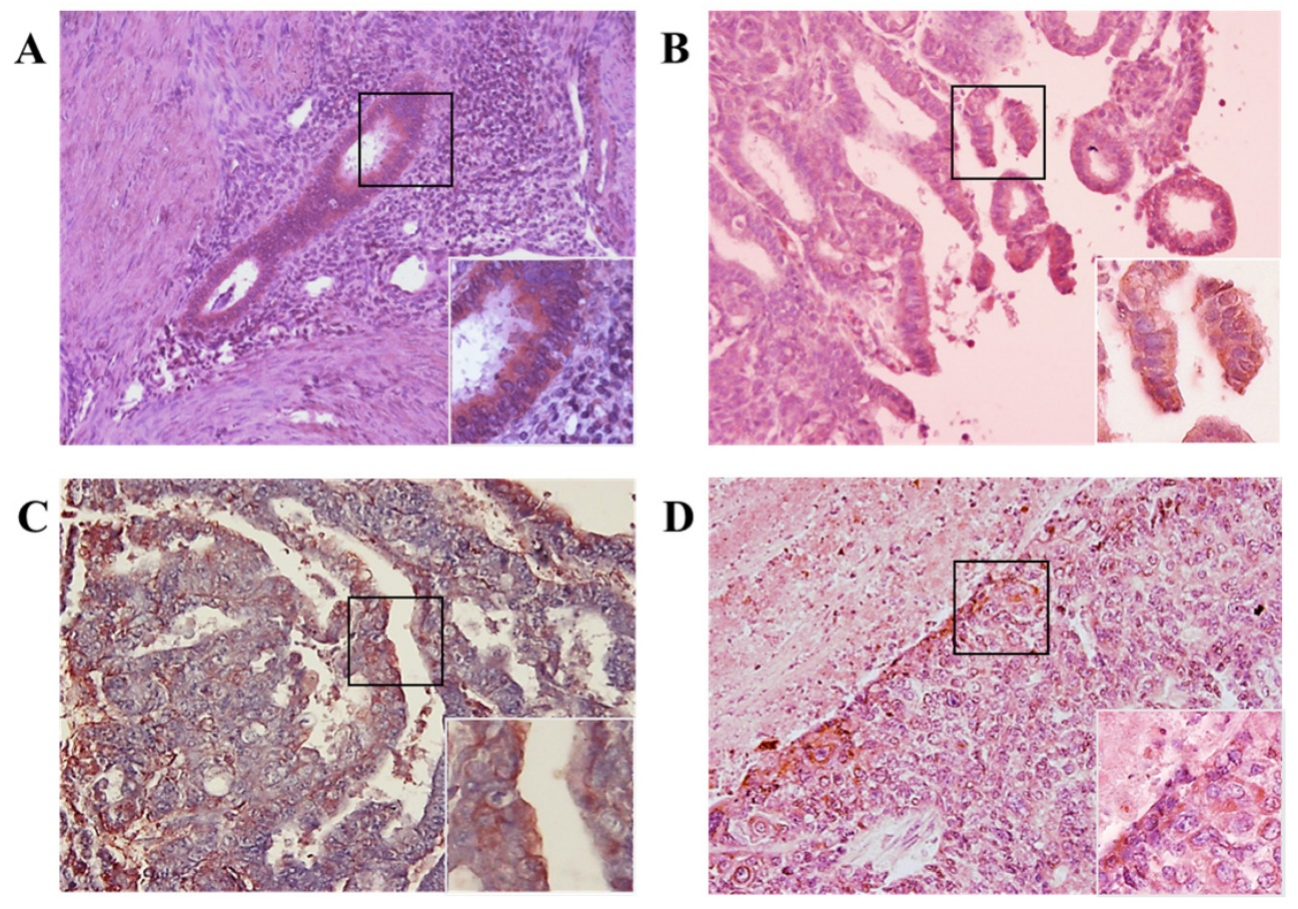
Light micrography of human proliferative endometrium (A) shows moderate expression for MIS/AMHRII in the cell membrane. In this and all subsequent panels, the right lower boxed area is higher magnification of the black boxed area (x400). Chromogen is AEC. Magnification, x200. (B) Well differentiated endometrioid adenocarcinoma moderately expresses MIS/AMHRII. (C) Moderately differentiated endometrioid adenocarcinoma strongly expresses MIS/AMHRII. (D) Poorly differentiated endometrioid adenocarcinoma moderately expresses MIS/AMHRII.

We investigated whether MIS/AMHRII protein is expressed in human endometrial cancer. Immunohistochemical staining was performed using rabbit polyclonal MIS/AMHRII antibody. All 10 human uterine endometrial cancer samples moderately or strongly expressed MIS/AMHRII protein in the cell membrane. In normal proliferative endometrium, endometrial cells showed moderate expression of MIS/AMHRII protein. Similarly, in well and poorly differentiated endometrioid adenocarcinoma, endometrial cancer cells showed moderate MIS/AMHRII protein expression. By contrast, moderately differentiated endometrioid adenocarcinoma cells strongly expressed MIS/AMHRII protein in the cell membrane (Fig. 1).

### Expression of MIS/AMHRII mRNA Detected by in situ Hybridization

**Figure 2:**
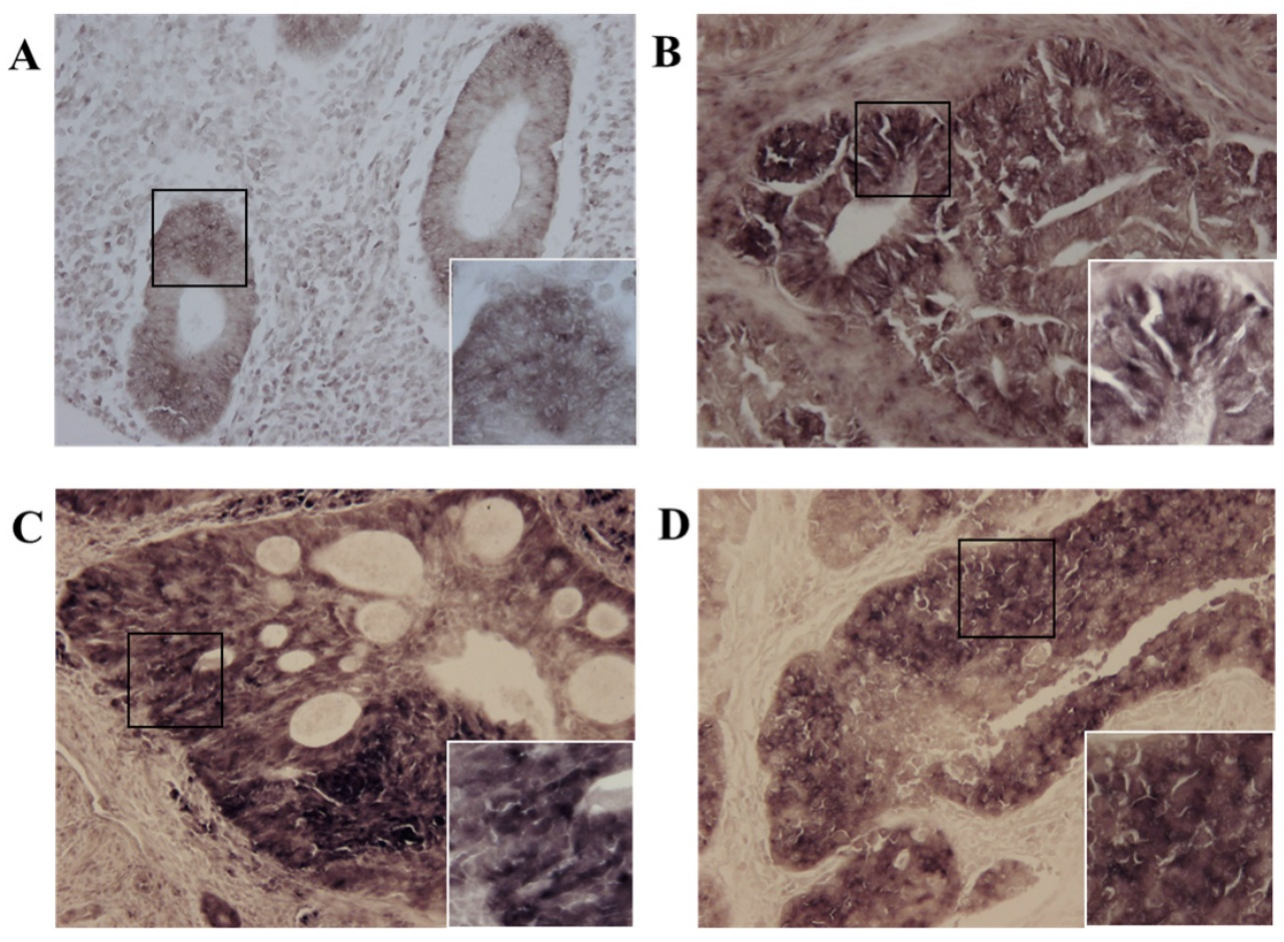
Light micrography of human proliferative endometrium (A) shows moderate expression MIS/AMHRII mRNA in the cell membrane. In this and all subsequent panels, the right lower boxed area is higher magnification of the black boxed area (x400). Chromogen is 4-nitroblue tetrazolium chloride/5-bromo-4-chloro-3- indolyl-phosphate (NBT/BCIP). Magnification, x200. (B) Well differentiated endometrioid adenocarcinoma strongly expresses MIS/AMHRII mRNA. (C) Moderately differentiated endometrioid adenocarcinoma strongly expresses MIS/AMHRII mRNA. (D) Poorly differentiated endometrioid adenocarcinoma strongly expresses MIS/AMHRII mRNA.

Next, we investigated whether MIS/AMHRII mRNA was expressed in human endometrial cancer. *In situ* hybridization was performed using the MIS/AMHRII RNA-probe. In all 10 human uterine endometrial cancer samples, cancer cells strongly expressed MIS/AMHRII mRNA in the cell membrane. The intensity of MIS/AMHRII mRNA expression did not differ according to differentiation. In normal proliferative endometrium, endometrial cells moderately expressed MIS/AMHRII mRNA. In well, moderately, and poorly differentiated endometrioid adenocarcinoma, the cancer cells showed strong expression of MIS/AMHRII mRNA in the cell membrane (Fig. 2).

### Endometrial Cancer Cell Culture

Except for two cases that do not have exponential growth, we proceeded our experiment with remaining three specimens. And the results were presented as an average of data from three specimens.

### Expression of MIS/AMHRII in Endometrial Cancer Cells Assessed Using Immunocytochemistry

**Figure 3:**
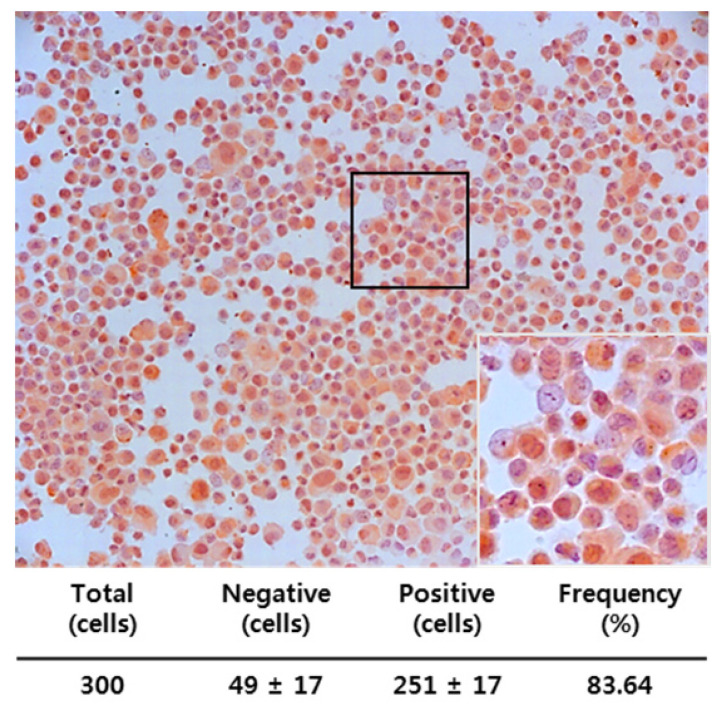
Endometrial cancer primary cultured cells express the MIS/AMHRII by immunocytochemistry with rabbit polyclonal anti-human MIS/AMHRII antibody. Chromogen is AEC. Magnification, x200. The right lower figure is higher magnification of the black boxed area (x400).

We also examined whether MIS/AMHRII, the target molecule for MIS/AMH, is expressed in human endometrial cancer cells. Immunocytochemical staining was performed using rabbit polyclonal MIS/AMHRII antibody. Of the cells examined, 83.64% (251/300) expressed MIS/AMHRII. A representative micrograph displaying the MIS/AMHRII staining of endometrial cancer cells is shown in Fig. 3. The average staining intensity score of five different endometrial cancer cell cultures was 2.80±0.45 (expression scores on a scale of 3: 3, 2, 3, 3, and 3).

### Effect of MIS/AMH on Endometrial Cancer Cell Viability

**Figure 4:**
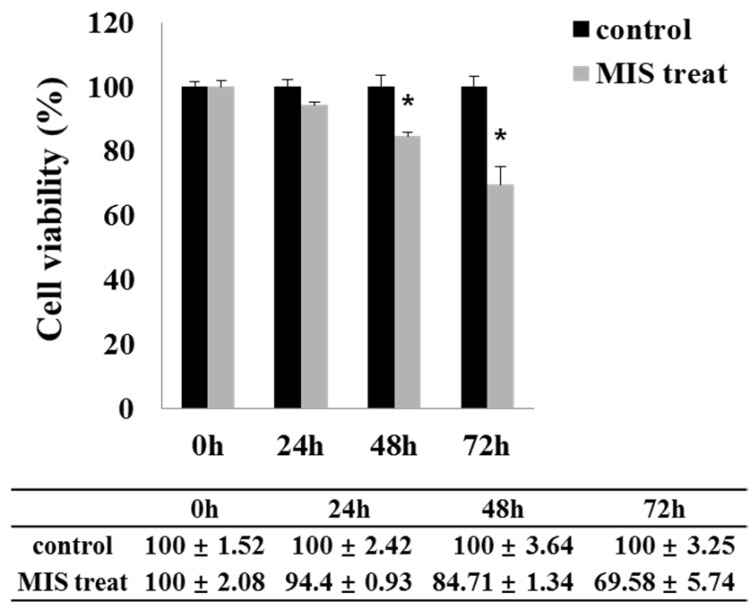
Effect of MIS/AMH on the viability of human endometrial cancer cells. Cells were treated with 10 μg/ml MIS/AMH for 24, 48 and 72 h, respectively. After treatment, cells were stained MTT, and the absorbance was read at 550 nm. Results were presented as percentage of control which was calculated using the equation: (mean absorbance of treated cells/mean absorbance of control cells) x100. Data were expressed as mean± standard deviation (SD). * *P*<0.05 as compared to corresponding control group, n=5.

MIS/AMH (10 µg/ml) exposure resulted in a significantly decreased viability of endometrial cancer cells. Upon MIS/AMH exposure, the viability of cells decreased by 94.4%, 84.71%, and 69.58% at 24, 48, and 72 h, respectively, relative to that of untreated control cells (Fig. 4). Endometrial cancer cells showed a negative correlation between MIS/AMH exposure time and cell viability (*P*<0.05).

### Effect of MIS/AMH on the Cell Cycle of Endometrial Cancer Cells

**Figure 5:**
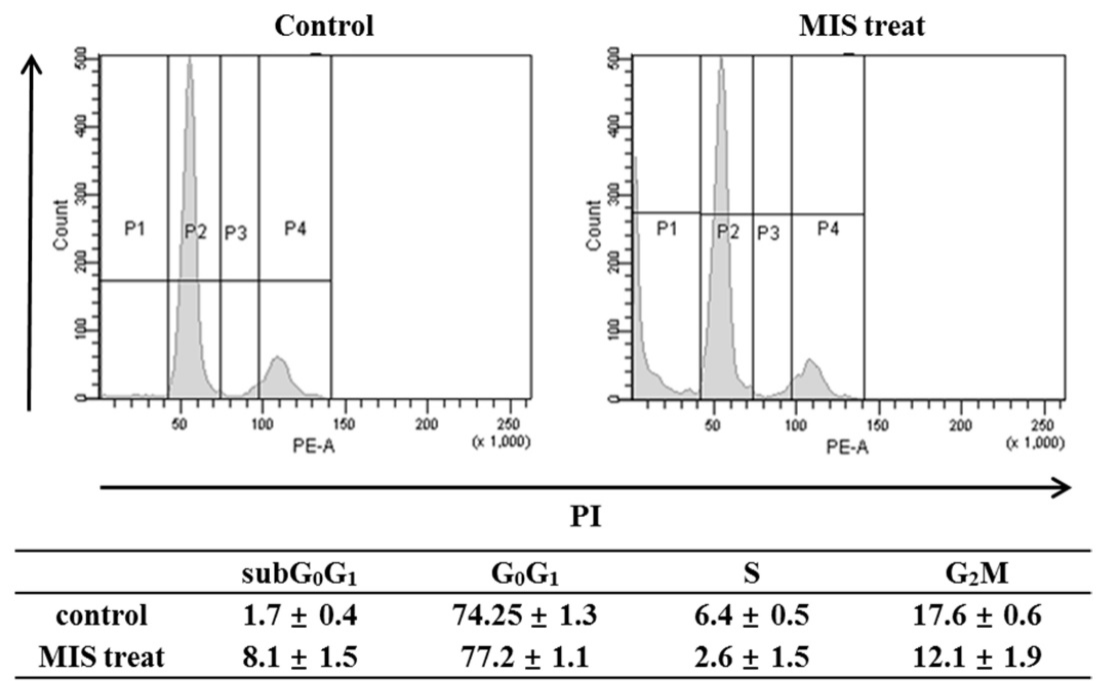
Cell cycle distribution after exposure to human endometrial cancer cells with 10 μg/ml MIS/AMH. Propidium iodide stain was performed and analyzed by flow cytometry. Histograms of cellular DNA content were obtained by flow cytometry. Data were expressed as mean± standard deviation (SD) from five independent experiments.

To determine whether the anti-growth activity was related to cell-cycle regulation, the cell cycle phase distribution after MIS/AMH (10 µg/ml) treatment was analyzed by flow cytometry. When cells were incubated with MIS/AMH for 72 h, the percentages of cells in the S and G_2_/M phases were decreased, whereas those of cells in subG_0_/G_1_ and G_0_/G_1_ phases were increased. After 72 h, the numbers of endometrial cancer cells in subG_0_/G_1_ and G_0_/G_1_ phases were increased by 8.1% and 77.2%, respectively, and those in S and G_2_/M phases were decreased by 2.6% and 12.1%, respectively (Fig. 5). This may indicate that MIS/AMH induces G_0_/G_1_ arrest, subsequently leading to cell death.

### Effect of MIS/AMH on the Apoptosis of Endometrial Cancer Cells

**Figure 6:**
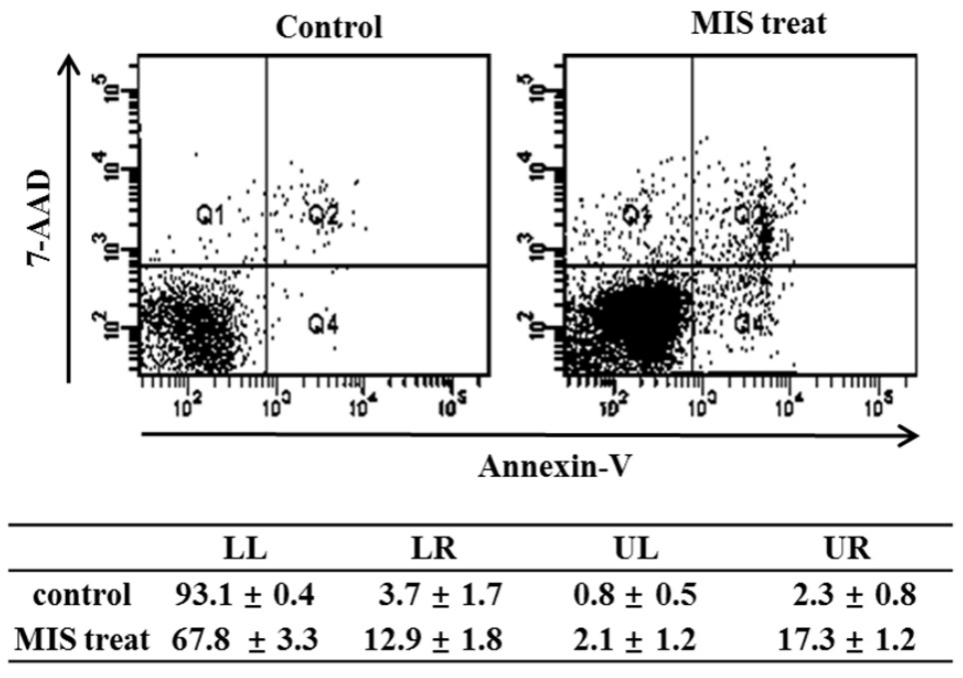
Apoptosis distribution after exposure to human endometrial cancer cells with 10 μg/ml MIS/AMH and externalization of phosphatidylserine assessed by measuring Annexin-V-FITC binding using 7-AAD as a counterstain (left lower quadrant, surviving cells; right lower quadrant, early apoptotic cells). Data were expressed as mean± standard deviation (SD) from five independent experiments.

To detect apoptosis in response to MIS/AMH (10 µg/ml) administration, the externalization of phosphatidylserine was assessed by measuring PE annexin V-binding using 7-AAD as a counterstain. The percentage of endometrial cancer cells undergoing apoptosis increased from 3.7% in control to 12.9% after 72 h of MIS/AMH exposure. The proportion of surviving cells, early apoptotic cells, and late apoptotic cells in the annexin-V staining was 93.1%, 3.7%, and 2.3%, respectively, in control and 67.8%, 12.9%, and 17.3%, respectively, after 72 h of treatment with MIS/AMH (Fig. 6).

### MIS/AMH-Induced Alteration of Regulatory Proteins in Endometrial Cancer Cells

**Figure 7:**
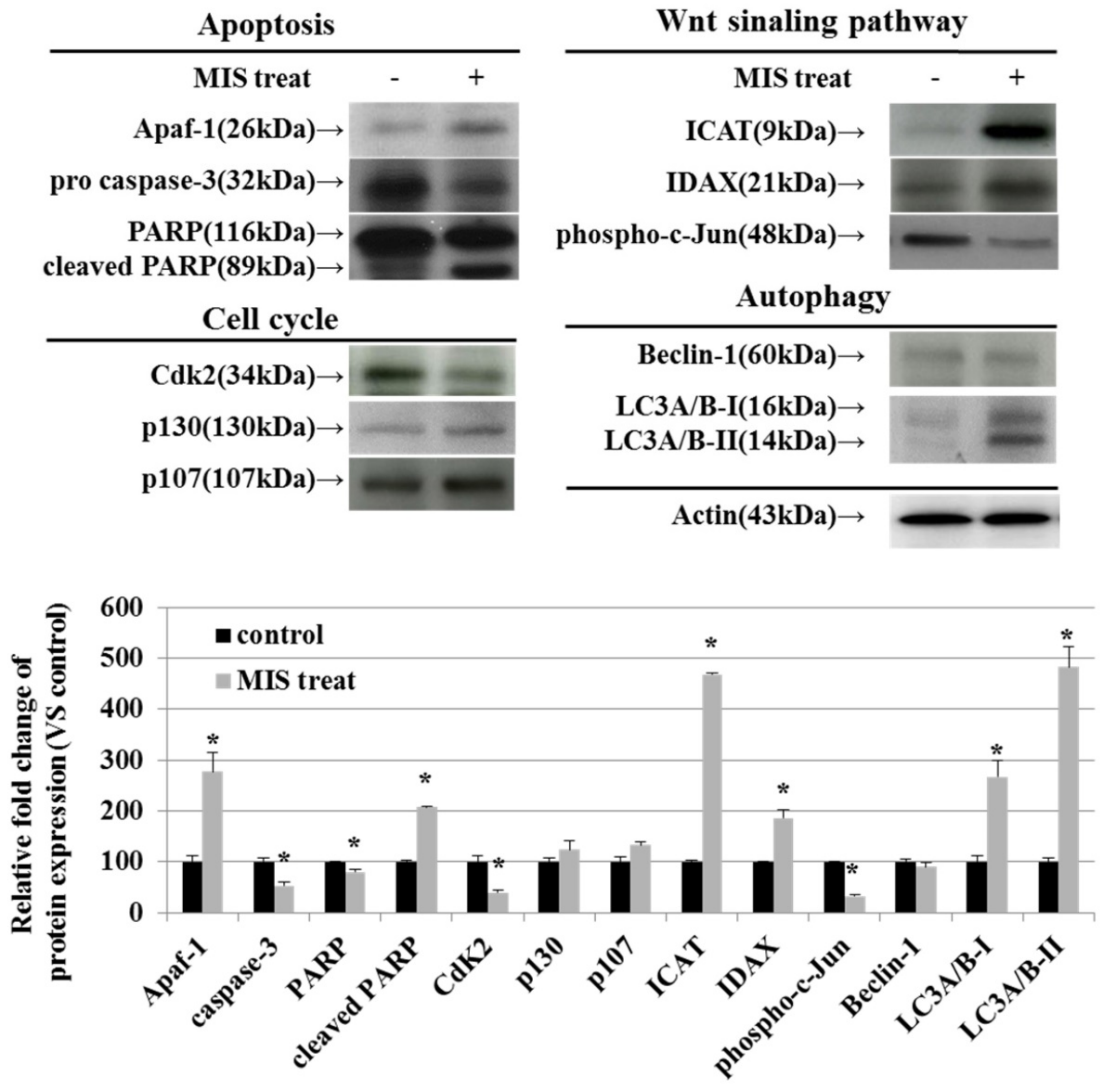
Western blot verification of apoptosis, cell-cycle, Wnt signaling pathway and autophagy-related proteins in human endometrial cancer cells treated with 10 μg/ml MIS/AMH. The protein expression data were shown as the mean ± SD. *P<0.05 compared with MIS/AMH untreated group, n=3 per group.

To identify the relevant signal transduction pathway used by MIS/AMH (10 µg/ml) in endometrial cancer cells, we performed western blotting on apoptosis-, cell cycle-, Wnt signaling pathway-, and autophagy-related proteins. Among apoptosis-related proteins, the levels of APAF-1 and cleaved poly (ADP-ribose) polymerase (PARP) were increased following MIS/AMH treatment. However, MIS/AMH treatment decreased the levels of pro-caspase-3 and PARP. Among cell cycle-related proteins, p130 and p107 levels were increased, whereas the levels of CDK2 were decreased by MIS/AMH treatment. Regarding Wnt signaling pathway-related proteins, MIS/AMH treatment increased ICAT and IDAX levels but decreased phospho-c-Jun levels. Among autophagy-related proteins, light chain 3 (LC3)A/B-I and LC3A/B-II had increased levels, whereas beclin-1 showed decreased protein levels following MIS/AMH treatment (Fig. 7).

## Discussion

MIS/AMH is a naturally occurring growth inhibitor that initiates its effects by binding to specific receptors. Since MISRII/AMHRII has been shown to be expressed in gynecologic cancers, MIS/AMH has been suggested as an effective adjuvant treatment for these cancer types. Previous studies have reported that MIS/AMH inhibits proliferation in tissues and cell lines of gynecologic malignancies such as ovarian cancer 9-13, cervical cancer 14, 15, and endometrial cancer 16, 17. It has also been suggested that MIS/AMH regulates cell proliferation of cervical, endometrial, and ovarian cancer cell lines by modulating cell cycle, apoptosis, and Wnt signaling pathways 9-17.

In cervical cancer, MIS/AMH suppresses cell division by inducing an increase in the expression of CDK inhibitors such as p16 and p107, which results in a decrease in the activity of the CDK complex, leads to inhibition of E2F activity, and induces G_1_ arrest and subsequent apoptosis 15, 20.

In the endometrial carcinoma cell line AN3CA, MIS/AMH reduces cell viability by regulating pathways associated with cell cycle arrest, apoptosis, and Wnt signaling 17. MIS upregulates p107 and p130 in AN3CA cells and is associated with cell cycle arrest due to a decrease in CDK2 levels. MIS/AMH induces apoptosis through activation of APAF-1 and PARP. It also reduces the expression of c-Jun, which is involved in MIS/AMH-induced tumor suppression by regulating ICAT and IDAX levels, which negatively modulate Wnt signaling pathways. It has also been reported that MIS/AMH exerts anti-cancer effects via apoptosis and Wnt signaling pathways. These are some of the known signaling pathways involved in the various MIS/AMH-mediated functions.

It has also been shown that in the human epithelial ovarian cancer cell lines OVCAR-8, MIS/AMH upregulates the expression of ICAT, which can modulate Wnt signaling. Increased ICAT expression interferes with the β-catenin/TCF/LEF-1 complex, preventing β-catenin from entering the nucleus. As a result, c-Myc and c-Jun activity decreases, and MIS/AMH-treated cells undergo apoptosis 12, 13.

Although MIS/AMH has been suggested as a promising candidate that may play a role in inhibiting gynecologic tumor growth* in vivo* and *in vitro*, its downstream signaling pathways have not been fully defined. Due to the lack of detailed genomic analyses, there are few targeted therapies for EC. In 2013, the Cancer Genome Atlas (TCGA) Research Network reported the integrated genomic characterization of EC 24. This highlighted a number of key pathways, suggesting a potential clinical role for various targeted therapies. According to the TCGA report, most endometrioid tumors expressed frequent mutations in *PTEN*, *CTNNB1*, *PIK3CA, ARID1A, and KRAS.* By contrast, serous tumors and high-grade endometrioid tumors had frequent *P53* mutations. The TCGA genomic EC profiles have highlighted several key pathways, one of which is the Wnt signaling pathway. Approximately 65% of endometrial cancers contain alterations within the Wnt signaling pathway 24.

Recent advances in genetic and molecular analyses of EC have led to the development of various targeted therapies. Trastuzumab, a human EGFR type II (HER2)-related inhibitor, has shown encouraging results in uterine serous carcinoma, which is characterized by overexpression of HER2 25, 26. Temsirolimus, which inhibits mTOR, has encouraging single-agent activity in endometrial cancer, irrespective of the *PTEN* status 27. Pembrolizumab, an anti-programmed death 1 monoclonal antibody, demonstrated a favorable safety profile and durable antitumor activity against MMR-deficient (dMMR) and MSI-high (MSI-H) tumors 28, 29. Lenvatinib, a multiple kinase inhibitor plus pembrolizumab, showed promising antitumor activity in patients with advanced endometrial carcinoma, regardless of the tumor MSI status 30, 31. However, there is still a need for new solutions to manage advanced, recurrent, and metastatic endometrial cancers 32. MIS/AMH has the potential as a therapeutic agent against endometrial cancer. Thus, it is important to better understand MIS/AMH-regulated mechanisms and genes.

The strength of our study is that we used patient-derived endometrial cancer cells, not an endometrial cancer cell line. In our study, we first confirmed the expression of MISRII/AMHRII in endometrial cancer tissues and cancer cells. This was confirmed by immunohistochemistry and mRNA *in situ* hybridization. We then examined the anti-proliferative effect of MIS/AMH on endometrial cancer cells. The results showed an approximately 30% reduction in cell survival, change in cell cycle parameters, and increase in cellular apoptosis. Finally, we used western blot analysis to confirm the relevant signal transduction pathway used by MIS/AMH.

p107 and p130 are known to regulate cell cycle progression through modulation of E2F transcription factor activity 33. In cervical cancer cell lines, induction of p130 and p107 by MIS/AMH appears to play an important role in the inhibition of proliferation 14. Our study showed slight upregulation of p107 and p130. However, the difference was not statistically significant. On the other hand, the expression of CDK2, a regulator of G_1_/S and S/G_2_ cell cycle transitions, was significantly reduced after MIS/AMH treatment. This result correlates with the observed cell cycle arrest and supports the hypothesis that MIS/AMH mainly acts via suppression of cell cycle activities by regulating CDK inhibitors and CDKs.

Apoptosis is a specific form of programmed cell death. It is responsible for eliminating damaged or unnecessary cells in multicellular organisms. APAF-1 is a key molecule in apoptosis 34. Our study showed a significant increase in APAF-1 levels after MIS/AMH treatment, indicating that MIS/AMH induces apoptosis in endometrial cancer cells.

Autophagy is an intracellular molecular pathway that maintains cellular homeostasis and is considered a key mechanism of cell death in endometrial cancer 35. In prostate cancer cells, autophagy is observed after blocking the Wnt signaling pathway 36. Similar results have been reported in breast cancer cells 37. In the present study, we found a significant increase in LC3, which is considered the most reliable biomarker of autophagy. These results suggest that MIS/AMH induces autophagy in endometrial cancer cells.

The Wnt signaling pathway is one of the most studied signaling pathways and is crucial in carcinogenesis and other oncogenic processes 38. Wnt signaling can be categorized into canonical (β-catenin-dependent) and non-canonical (β-catenin-independent) pathways. The majority of Wnt signaling aberrations in gynecologic malignancies occur in the canonical pathway 39.

β-catenin plays an important role in signaling transduction, including MIS/AMH signaling. The MISRII/AMHRII expression correlates both with the β-catenin cytoplasmic and nuclear accumulation pattern. After binding to its receptor, MIS/AMH induces an accumulation of β-catenin, which may interact with the transcriptional activators TCF and LEF in the nucleus and activates transcription of target genes 40. And Wnt target genes, including cyclin D1 and c-Myc are one of them 41. Thus, aberrant activation of this pathway causes the accumulation of β-catenin in the nucleus and promotes the transcription of many oncogenes. Many studies evaluated the role of Wnt signaling pathways in gynecologic malignancies 42-45. In endometrial cancer, hyperactivation of the Wnt signaling pathway seems to play a role in the development and progression of cancer 40. For example, mutations in the *CTNNB1* gene result in the accumulation of β-catenin protein and upregulation of the Wnt pathway 43.

In the present study, we investigated whether MIS/AMH mediates the regulation of Wnt signaling pathways. We found a significant increase in ICAT and IDAX expression, whereas phospho-c-Jun levels were significantly decreased after MIS/AMH treatment. Both ICAT and IDAX inhibit Wnt signaling pathways 46, 47. A previous study revealed that in Wnt signaling, c-Jun functions as a scaffold in the β-catenin TCF4 transcription complex 48. c-Jun transcriptional activity is stimulated by phosphorylation at two N-terminal sites 49. Thus, a decrease in phospho-c-Jun could result in tumor suppression through regulation of the Wnt signaling pathway. The Wnt signaling pathway has been discussed in several studies as a future target for personalized therapies of gynecologic cancers 50, 51. Our study indicates that the Wnt signaling pathway is part of the intracellular mechanisms downstream of MIS/AMH.

The findings of this study support the hypothesis that MIS/AMH was able to function as a potential therapeutic for endometrial cancer by targeting molecular pathways. However, before the hypothesis could be tested, one of the major challenges is how to deliver the right MIS/AMH dose at the tumor sites. No study has been reported a stable delivery system of MIS/AMH into tumor cells. And further studies are required.

In conclusion, MIS/AMH inhibits the growth of endometrial cancer cells via modulation of the autophagy, cell cycle and apoptosis. MIS/AMH also blocks the Wnt signaling pathway, suggesting its potential as a therapeutic agent in endometrial cancer.
